# Effect of Organics on Heavy Metal-Contaminated River Sediment Treated with Electro-Osmosis and Solidification/Stabilization Methods

**DOI:** 10.3390/ma13061466

**Published:** 2020-03-23

**Authors:** Chonggen Pan, Keyu Chen, Danting Chen

**Affiliations:** 1 Ningbo Institute of Technology, Zhejiang University, Ningbo 315100, China; 2 Ningbo Research Institute, Zhejiang University, Ningbo 315100, China; 3 School of Civil Engineering and Architecture, Zhejiang Sci-Tech University, Hangzhou 300018, China; chenkeyu0209@163.com; 4 Civil Environmental and Geomatic Engineering, University of College London, London WC1E 6BT, UK; 13269672200@163.com

**Keywords:** river sediment, fulvic acid, heavy metal, ion migration, strength

## Abstract

This study focuses on the treatment of heavy metal ions and achieving enhancement of river sediment, which is rich in organics. Fulvic acid was used as the main representative of organics in which to study the transfer of Cu^2+^, Zn^2+^ ions in the electroosmotic system, in both the absence and presence of organics. In addition, the effects of the experiment parameters (i.e., voltages, displacement, and water content), heavy metal ion content (0.19% and 0.38%, respectively), and the concentration of organics (1.5%, 3%, and 4.5%) acting on migration of ions and physiochemical properties of sediment, before and after electro-osmosis treatment were investigated. Mineral composition of the soil and its microscopic characteristics were analyzed by scanning electron microscopy. The results show that the 4.5% fulvic acid added in the sediment can enhance the migration ability of Cu^2+^ and Zn^2+^ ions through complexation, and simultaneously effectively reduce the formation of colloids with the average reductions of Cu^2+^ ions and Zn^2+^ ions being 28 and 11 mg/kg, respectively. While the dewatering capacity of the sediment with higher fulvic acid content is weaker, fulvic acid can effectively reduce the corrosion of the electrode during the electro-osmosis process, due to the film formed on the metal surface. Moreover, the fulvic acid in the organics can be combined with the aluminum and calcium produced by the hydration of the cement, delaying the hydration of the cement, while simultaneously decomposing the hydration product and hindering the hardening of the cement, thereby affecting and destroying the formation of the sediment’s structure and its strength.

## 1. Introduction

Dredged sediment is a type of slurried waste dredged from the bed of rivers, lakes, estuaries, or coastal waters, usually featuring low strength and high compressibility. The dredging activities are normally performed to meet navigational needs or remove the contaminated deposits. With the rapid growth of Chinese industries, the annual production of sediment in China is up to 300 million tons and the number will continue to increase as reported [1,2]. The sediment, normally composed of fine particles, can adsorb potential contaminants such as nutrient elements and heavy metals, when present in excessive amounts. The contaminants bound to sediment can also be released to a water body, causing the degradation of water quality [3]. Recent examples include the contaminated sediment found in Qiandao Lake in China [4], Poyang Lake in China [5], the Zihu River Basin in China [6], the Tehran Basin in Iran [7], the Ganges in India [8], Ichkeul Lake in Tunisia, and the Axios and Aliakmon basins in Greece [9]. The contaminants in these waters are heavy metals (i.e., Cu, Zn, Cr, Ni, Cd, and Pb) and organics (i.e., humic acid and fulvic acid), which are severe threats to the aquatic system in the regions [10,11].

Various methods have been used to treat dredged sediment contaminated with heavy metals [12], which include the electro-osmosis method, the phytoremediation method, the chemical leaching method, and the solidification/stabilization method, etc. [13]. The solidification/stabilization (S/S) method is an in-situ or ex-situ remediation method that has been widely used for Brownfield lands and Superfund sites. By adding proper chemical agents to contaminated soils, S/S can enhance the soil strength while immobilizing the contaminants inside the stabilized soils. The treated soils can be landfilled, reused on site to support redevelopment, or even recycled off site as construction materials. The chemical binders used are mainly cementitious materials (cement, fly ash, lime) and sodium silicate [14,15,16]. For the sediment contaminated by both heavy metals and organics, some conventional cementitious binders may no longer work, as organics can impede the cement hydration. For example, humic substances and fulvic acid can combine with calcium and aluminum from cement to reduce the supply of calcium and aluminum for hydration, and meanwhile disturb the alkaline environment, which therefore compromises S/S effectiveness for heavy metal encapsulation [17,18]. Moreover, when the sediment contains a significant amount of unfavorable heavy metals (e.g., Zn^2+^, Pb^2+^, and Cu^2+^), cement hydration would be retarded. Organics such as fulvic acid can be bound to some potentially toxic elements (PTEs), such as Pb, Cd, Cu, and Cr through complexation, which results in the coexistence of heavy metals and organics [19,20,21]. Overall, a single method of S/S may not be adequate for the remediation of the sediment containing both heavy metals and organics, and the combined use of two or more methods may be necessary to achieve satisfactory results. The electro-osmosis method is useful in accelerating the consolidation of soft soils by applying electric field to the soils to cause motion of water. As compared with other methods, this method possesses the advantages of a high treatment efficiency, low environmental impact, high efficiency for remediating organic contaminants, and high remedy efficiency for small-area sediment [22,23,24,25,26,27]. The underlying mechanisms of the electro-osmosis method in remediating contaminated sediment involve [28]: electro-osmosis, electromigration, electrophoresis, convection, and dispersion. For ionizable inorganic contaminants, their motion in the electric field is mainly controlled by electromigration at high contaminant concentrations, and by electro-osmosis at low contaminant concentration [29]. However, the adsorption and desorption process in sediment can alter the contaminant concentration, which results in the contaminant migration being dominated by electromigration. In all, the contaminant concentration is a key parameter in the electro-osmosis process, which will be studied in this study.

The adsorption retention capacity of heavy metal ions is dependent on ion species, ionic strength, pH, soil cation exchange capacity (CEC), organics, temperature, soil particle size, etc. [29,30,31,32]. The migration of heavy metal ions under electro-osmosis treatment has been studied by many researchers [33], who added citric acid (CA), nitric acid, and hydrochloric acid to several clay specimens containing various heavy metals during electro-kinetic remediation, and found that acidic electrolytes could effectively reduce the adsorption of heavy metals. Amrate [34] applied the electro-kinetic remediation for the EDTA-contaminated clays, and found that the chelating agent could significantly lower the heavy metal concentration by means of complexation. Liu [35] investigated the effects of heavy metal concentration, water content, and other factors on the binary competitive adsorption characteristics of Pb(II), Cu(II), and Cd(II) in loess using various isothermal adsorption models. They confirmed that the adsorption capacity in multiple heavy metal systems was lower than in a single heavy metal system. As the initial heavy metal concentration increased, the optimum adsorption capacity for the three heavy metals on the unit loess was increased. The above studies are primarily focused on the migration mechanism of heavy metal ions. However, limited studies are focused on remediation effectiveness for both organics and heavy mental using the electro-osmosis method.

The objectives of this study were twofold: (1) to investigate the effect of organics (fulvic acid) on the migration of heavy metal ions (Cu^2+^, Zn^2+^) in the sediment treated by the electro-osmosis method, (2) to evaluate the effect of organics on the mechanical properties of the sediment treated by the S/S method. In the electro-osmosis tests, the Voltage and pH values were varied to assess the changes in heavy metal concentration and mechanical properties of sediment with various organics contents. In the S/S tests, the unconfined compression strength (UCS) test, a scanning electron microscope (SEM) test, and an X-ray deflectometer (XRD) test were conducted to elucidate the influence of organics contents on the strength and microstructure of solidified sediment.

## 2. Materials and Methods

### 2.1. Material Properties

**Figure 1:**
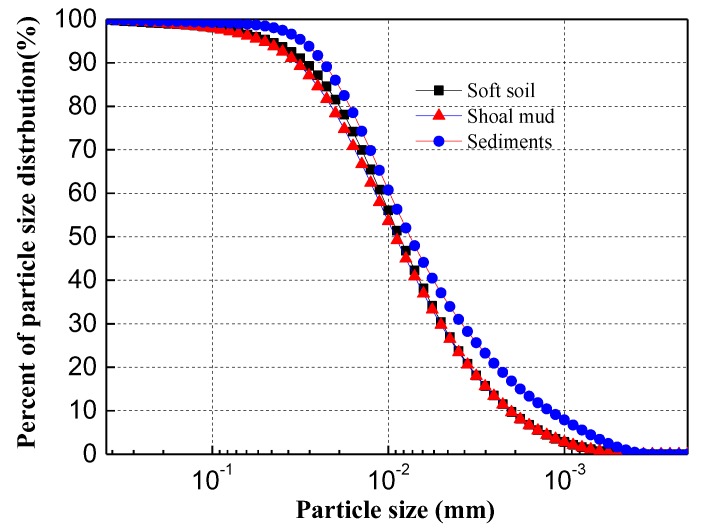
Size distribution of river sediment.

The sediments used in the experiment were taken at depths of 0.3 to 2 m below the bed of the Qiantang River in the city of Ningbo, China. They were in the form of slurry, composed of sediment clays in a grey to black color, and with an organic (i.e., fulvic acid) content of 2.5%. The particle size distribution of the sediment (size range: 0.3~5000 nm), which was determined using a Malvern Zetasizer NanoS90 laser particle size analyzer (Malvern, UK), is presented in Figure 1. For comparison purposes, Figure 1 also includes two typical soft soils in Ningbo. The test sediment shows the similar particle size distribution pattern to the typical soft clays in the region, but has a slightly higher fines contents. The clay fraction (≤2 µm) of the sediment accounts for 23%. 

The other basic properties of the sediment are summarized in Table 1, which were obtained based on the dried sediment (65 ± 2 °C) mixed with 68% water. These tests were conducted according to the standard for geotechnical testing (GBT50123-1999) and the standard for soil environmental quality (GB15618-1995).

Since the initial organics content in the sediment was relatively small, to evaluate the effects of organics on the effectiveness of electro-osmosis and solidification/stabilization (S/S), fulvic acid was used to represent organics and added to the sediment. For the solidification/stabilization method, the binder used was Portland cement with a 28-day compressive strength of 32.5 MPa.

### 2.2. Testing Methods

#### 2.2.1. Electro-Osmosis Experiment

**Figure 2:**
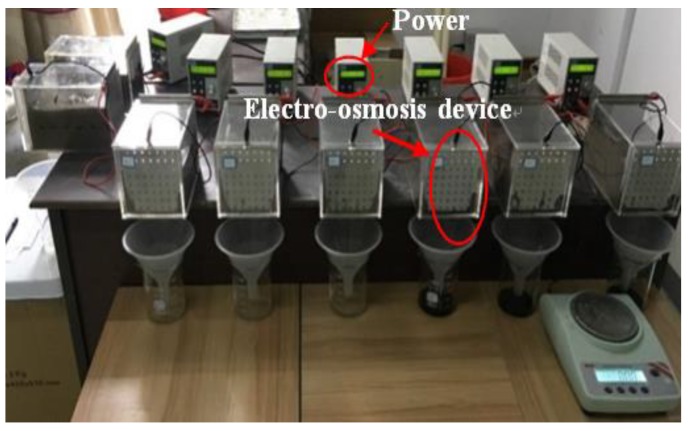
Setup of electro-osmosis test.

The electro-osmosis device was installed as in Figure 2. It mainly comprised of a test tank (180 × 120 × 130 mm), a metal electrode (120 × 130 × 3 mm), and power. According to the characteristics of the electroosmotic migration of pollutants in the soil [28,31], the stability of electro-osmosis and the same displacement of the electro-osmosis terminal point in the early stage are controlled by the constant current stability.

In addition, according to the investigation of the sediment characteristics of a river section heavily polluted by heavy metals in Ningbo city of China, the organics content was found to be about 7%. In the electro-osmosis experiment, 4.5% fulvic acid was added into the natural sediment, with an initial organics content of 2.5%. The test design is shown in Table 2.

Among them, Fulvic acid refers collectively to a set of organic acids, natural compounds, and components of the humus (CAS No. 479-66-3). They share a similar structure with humic acids, with differences being the carbon and oxygen contents, acidity, and degree of polymerization, molecular weight, and color. Fulvic acid is widely found in soil and water bodies (such as rivers, lakes, oceans, groundwater, etc.) and sediment such as coal. In addition, fulvic acid is still a complexion agent with strong complexion ability, according to the complex reaction:

M(H_2_O)_n_ + L = M(H_2_O)_n−1_L + H_2_O (1)

where M represents a metal ion; M(H_2_O)_n_ represents free metal ions in the solution, namely hydrated ions. L stands for fulvic acid molecule or charged ion; M(H_2_O)_n−1_L represents the complex that carries the metal ions, namely the coordination compound, and H_2_O represents the water molecule.

Equation (1) shows that the charged ion fulvic acid has a hydroxyl group, a carboxyl group and the likely complexes with a metal ion carried by a free water group, and the water group are released in the form of water (H_2_O) by removing the metal ion. The acid-substituted water group forms a fulvic acid complex with the metal ion for deposition.

**Figure 3:**
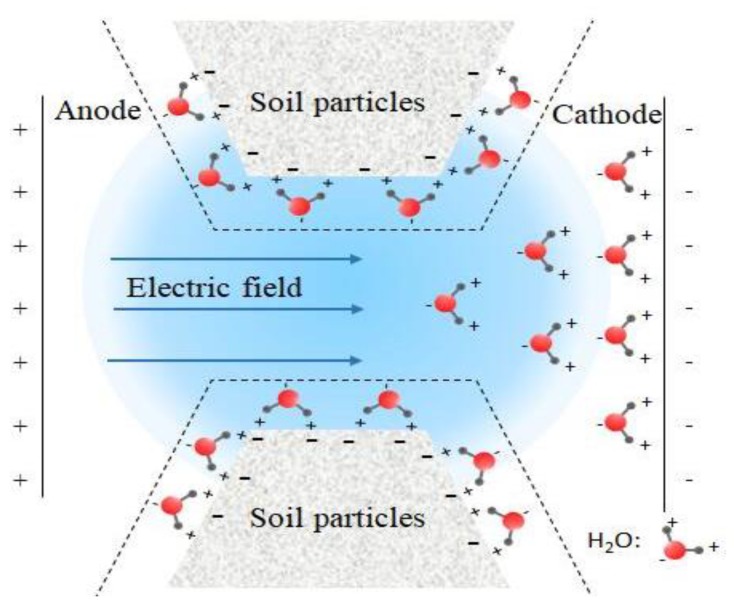
Electroosmosis principle of soft soil.

As shown in Figure 3, the dewatering of electro-osmosis plays an important role in the reinforcement of soft soil. Under the action of the current electric field, polar water molecules discharge from cathode. The reinforcement effect of the electro-osmosis method on the soft soil foundation mainly includes the acceleration of the dewatering of pore water in soil, the aggregation of soil particles near the anode, or the filling and densification of pores by colloidal products [36]. Shen et al. [37] believed that the decrease of soil moisture content improves its shear strength and cohesion, which is also the main reason for the increase in strength of soft soil after the dewatering of electro-osmosis. In addition, the negatively charged soil particles move toward to the anode under the action of the electric field, which reduces the porosity of the soil near the anode, and increases the strength of soft soil. Meanwhile, a series of chemical reactions took place near the electrode during electro-osmosis [38,39,40,41].

#### 2.2.2. Unconfined Compressive Strength (UCS) Experiment

An EBC-1000 microcomputer bending test instrument (Beijing, China) was used to carry out the unconfined compression strength (UCS) test. For different groups, an average of three strengths was used as the result of UCS. The experiment followed the procedure described in ‘JGJ/T 233-2011 Specification for mix proportion design of cement soil’. Following the UCS testing of each sample, adequate soil material in the middle of the cube sample was collected and dried for lead leaching and SEM tests.

#### 2.2.3. Microscopic Analyzes

To investigate the effect of stabilization, a SEM analyzer Quanta 650FEG (FEI, Waltham, MA, USA) and an X ray diffraction X’Pert PRO analyzer (PANalytical, Almelo, The Netherlands) were adopted to analyze the microscopic characteristics of the samples. Meanwhile, samples prepared for the test were milled and refrigerated, while those prepared for SEM testing were kept in absolute ethanol to prevent further reactions.

## 3. Results and Discussion

### 3.1. Effect of Parameters Change of Electro-Osmosis

**Figure 4:**
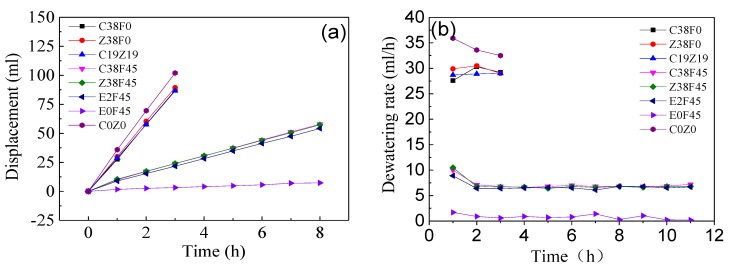
Displacement and drainage rate change of river sediment. (**a**) Displacement of river sediment; (**b**) Dewatering rate of river sediment.

Figure 4 shows that electro-osmosis was carried out by means of a constant flow of electricity, and the dewatering rate of different groups basically remained stable, while the concentration of heavy metals and volume of the solution were controlled at the same level, comparing the displacements and dewatering rates of different groups. Figure 4a shows that the displacements of groups C38F0, Z38F0, and C19Z19 are 87.1, 89.4, and 86.6 mL, respectively, and the displacements of groups C38F45, Z38F45, and E2F45 are 78.6, 77.7, and 73.9 mL, respectively. Meanwhile, as shown in Figure 4b, the dewatering rates of groups C38F0, Z38F0, andC19Z19 is about 29 mL/h. It could be concluded that the types of heavy metals have little effect on the displacement and dewatering rate. At the same time, comparing the dewatering rates of groups C38F0 and C38F45, Z38F0 and Z38F45, C19Z19 and E2F45 doped with fulvic acid or not, it was found that C38F45 decreases by about 77.6%, Z38F45 decreases by about 78.5%, and E2F45 decreases by about 78.2%, which indicates that the addition of an electrolyte under a constant current caused the decrease of the dewatering rate, and the effect of fulvic acid was contrary to that of chemical solutions (i.e., phosphate, aluminiumions, and phosphoric acid). It was found from the literatures [42,43] that the injection of chemical solutions during electro-osmosis were effective in enhancing the electro-osmosis and increasing the undrained shear strength of soil. Besides, the effect of electro-dewatering is related to the electrode types, electrode arrangement, and electrified methods.

As the reference group, the dewatering rate of group E0F45 remained at a stable and low level under 1.5 mL/h in the condition of no electricity, which indicates that the gravity field still plays a certain role in simulating one-dimensional horizontal dewatering electro-osmosis. In addition, it was observed during the experiment that the plasticity of the sediment became stronger after adding fulvic acid.

### 3.2. pH and Water Content of Sediment with Electro-Osmosis Treatment

**Figure 5:**
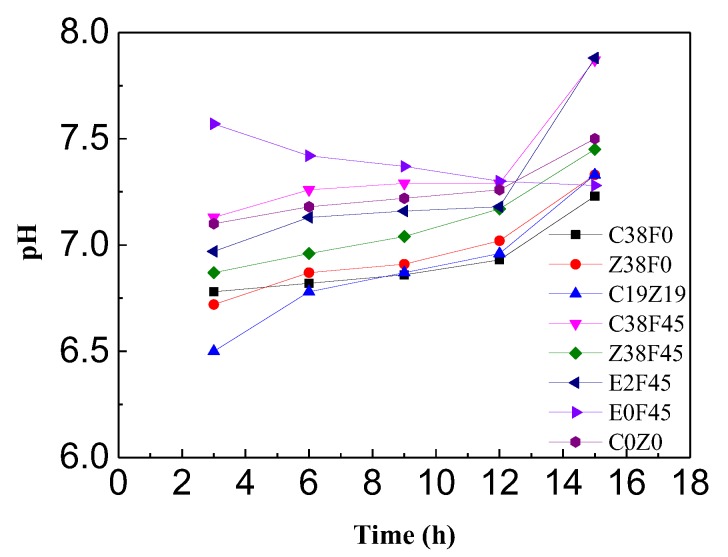
pH changes of different groups.

The red dot line in Figure 5 is the pH value (7.25) of the sediment before electro-osmosis treatment. The previous results show that the decrease of the pH value is beneficial to the dissolution of metals into water [44]. In this study, group E0F45as the control group without being energized, and the pH remains at the original value (7.25) after 15 h. Except for group E0F45, the pH values in different groups tended to increase near the cathode, which was caused by a redox reaction in the soil under the condition of electrification, generating H^+^ ions near the anode (Equation (2)) and OH^−^ ions near the cathode Equation (3):

M + nH_2_O = M(OH)_n_ + 2nH^+^ (2)

H_2_O + 2e^−^ = 2OH^−^ + H_2_↑ (3)

where, M represents a metal ion, M(H_2_O)_n_ refers to a free metal ion in the solution, that is, a hydrated ion.

**Figure 6:**
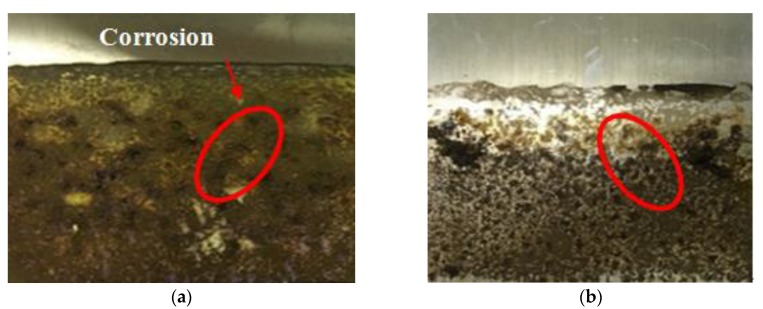
Electrode corrosion (**a**) Sample with no fulvic acid; (**b**) Sample mixed with fulvic acid).

After electro-osmosis treatment, the electroosmotic cathode showed no corrosion, while the electroosmotic anode was corroded to different levels. In addition, the electroosmotic anodes of different groups (Figure 6a), which is not doped with fulvic acid, produced a plurality of corrosion pits, whereas the groups of electroosmotic anodes (Figure 6b) doped with fulvic acid had less, indicating that fulvic acid can effectively reduce the corrosion of the electrode during electro-osmosis, which is different from the accelerated corrosion results reported in the literature [45].The main reason causing this phenomenon is that a high concentration of humic acid may be adsorbed on the surface of the electrode, and react with corrosion products and iron oxides, resulting in a dense adsorption film being formed on the metal surface, which can block the diffusion behavior of corrosion ions and reduce the corrosion rate of the electrodes to some extent.

**Figure 7:**
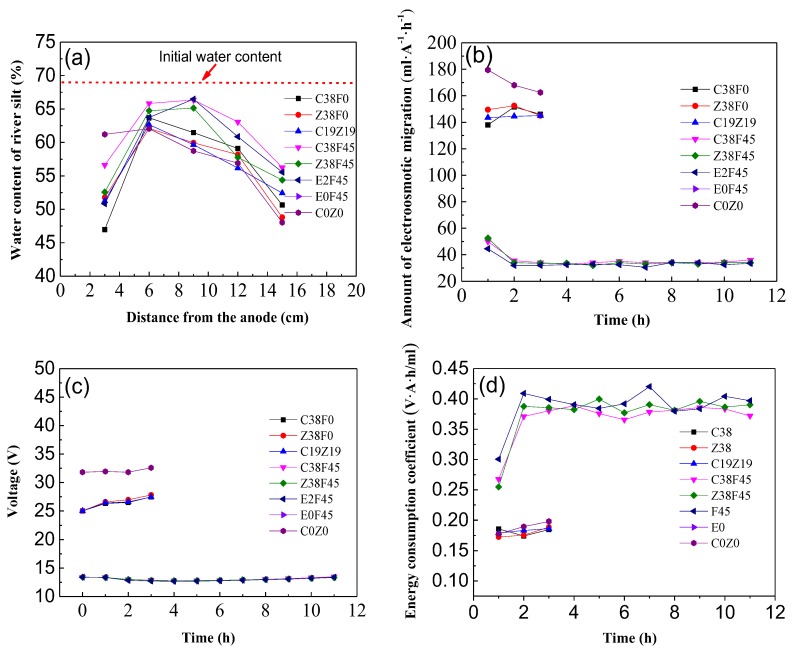
Effects of electro-osmosis treatment on various groups of river sediment. (**a**) Water content; (**b**) Amount of electroosmotic migration; (**c**) Voltage; (**d**) Energy consumption coefficient.

As shown in Figure 7a, the initial water content of natural sediment is 68%. In the case of similar displacement, the final moisture content of the group C0Z0 after the end of electro-osmosis showed a downward trend from anode to cathode: when the distance from the anode reached 15 cm, the final water content dropped to 48.0%, and the final water content of the other groups showed the same trend. The anode to cathode flow showed a trend of increasing firstly and then decreasing, which is different from the results of most electro-osmosis studies [42,43,44,45]. This may be due to the influence of the externally mixed electrolyte on the conductivity, which in turn causes the change of current, thereby affecting the electro-osmosis process.

### 3.3. Variation of Electro-Osmosis Migration, Voltage and Energy Consumption Coefficient

Figure 7b shows that the migration amount (MA) of electro-osmosis remained stable in the early stage, and the electro-osmosis migration amount of groups C38F0, Z38F0, andC19Z19 and C0Z0 was much higher at the initial stage of electro-osmosis. In addition, under constant current, the incorporation of electrolyte results in a lower MA, and the MA values of the electro-osmosis group with the same amount of electrolyte are identical, which is consistent with the assumption of H-S theory, indicating that the MA is affected by soil conductivity.

The migration amount is an important indicator for measuring the effect of electro-osmosis [46,47,48,49,50]. Li [49] found that the salt content of electrolyte has an influence on the electroosmotic behavior of soft clay, and migration helps to obtain optimal content in this process. Tao [50] proved that the sensitivity of electroosmotic migration to soil moisture content is closely related to soil type, and the sensitivity of the same soil may be greatly different with a different water content range.

As shown in Figure 7c, by comparing the voltage changes of C38F0, Z38F0, and C19Z19 (or C38F45, Z38F45, and E2F45) corresponding to the displacement and dewatering rate, it can be found that the equivalent voltage is not affected by the type of heavy metals under the condition of constant current electricity. At the same time, the equivalent voltage of groups C38F0, Z38F0, and C19Z19 showed an overall increasing trend, and the average voltage increased by 10.3%, while the equivalent voltage of groups C38F45, Z38F45, E2F45, and C0Z0 remained in a stable state of about 13.4 V. It maybe that the addition of fulvic acid coating on the surface of soil particles, and the pore passage of the soil being blocked, greatly slowed down the dewatering rate of constant current electricity, resulting in the initial electro-osmosis of groups C38F45, Z38F45, E2F45, and C0Z0.

Figure 7d shows that the average energy consumption coefficient of group A (C38F0, Z38F0, and C19Z19) in the early stage of electro-osmosis is only 0.18 VAh/mL, and the average energy consumption coefficient of group B (C38F45, Z38F45, and E2F45) is 0.37 VAh/mL, while the average equivalent voltage of group A is twice as much as group B, indicating that the energy consumption coefficient of the electroosmotic process is controlled by the equivalent voltage, regardless of the type of electrolyte.

Nowadays, new electrode materials like electro-technical synthetic materials (EKG) are investigated in order to improve dewatering and prevent electro-osmosis during the process of electro-osmosis [51,52]. However, its interface resistance is higher than that of metal, and the energy consumption is relatively larger; moreover, more researchers focus on the charged nanoparticles [53] and adjusting the energizing and pressurizing conditions to achieve the preset effect of dewatering and energy reduction [54].

### 3.4. Effect of Organics on Heavy Metal Ion igration

In order to investigate the migration of heavy metals by adding fulvic acid under dewatering electro-osmosis, the total amount of heavy metals in the sediment was analyzed for each set of measuring points from different positions of anode and electrode.

**Figure 8:**
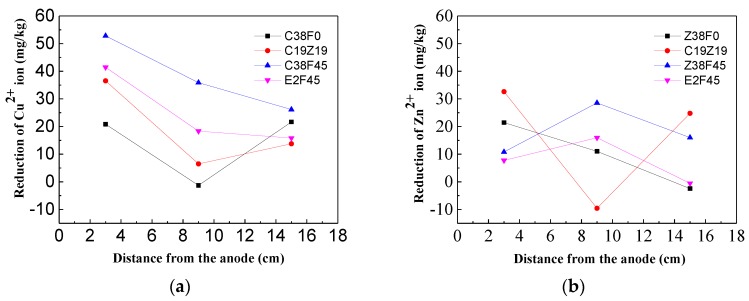
Reduction of Cu^2+^ and Zn^2+^ ion in sediment. (**a**) Reduction of Cu^2+^ ion; (**b**) Reduction of Zn^2+^ ion.

**Figure 9:**
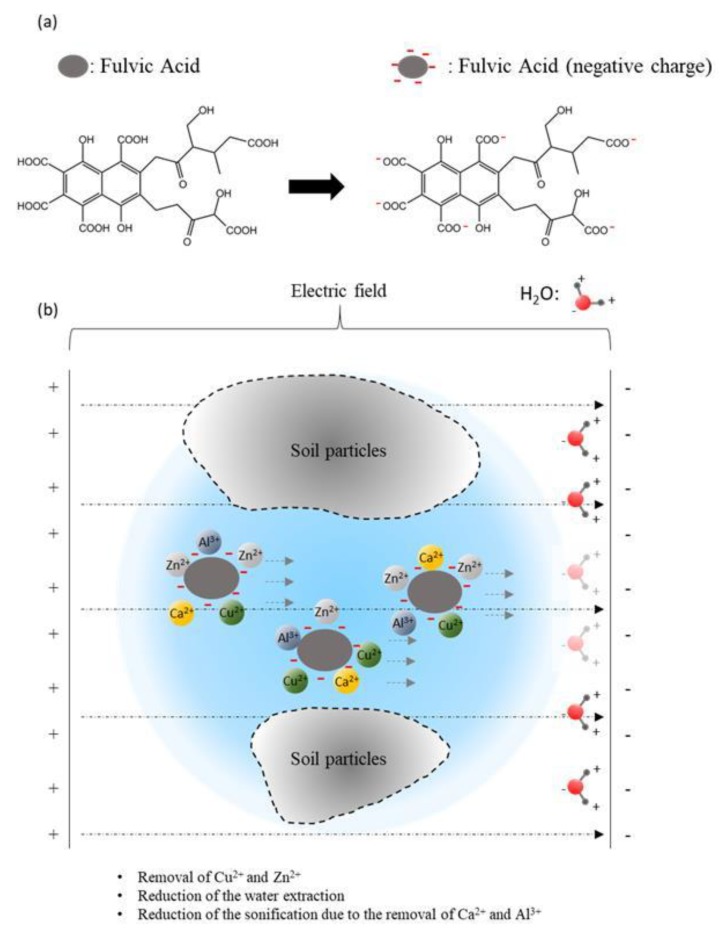
(**a**) The tendency of the negative charge of fulvic acid; (**b**) influence of the fluvic acid on the solidification of the sediment.

Figure 8 shows that the migration of Cu^2+^ ions and Zn^2+^ ions from the anode electrode to the cathode electrode generally decreases, with the average reductions of Cu^2+^ ions and Zn^2+^ ions being 28 and 11 mg/kg, respectively. It indicates that the migration ability of Cu^2+^ ions under electro-osmosis is stronger than that of Zn^2+^ ions. At the same time, the average ion migration rate (migration amount/total amount) of Cu^2+^ ions and Zn^2+^ doped with a fulvic acid group is 9.2% and 4.2%, respectively, and the non-doped fulvic acid group is 4.2% and 1.3%, respectively, which indicates that fulvic acid could effectively reduce the adsorption between heavy metal ions as well as soil particles, and increase the migration of heavy metal ions. For the Cu^2+^ content changes of groups C38F0 and C19Z19, C38F45 and E2F45, it was found that the Cu^2+^ reduction of groups C38F0 and C38F45 reached a minimum in the centre, between two electrodes. It may because heavy metal ions and OH^−^ formed colloid, which greatly reduces the pore stranded anode, and causes poor dewatering in the centre of electro-osmosis equipment. Meanwhile, the complexation of fulvic acid can effectively reduce the generation of the colloid as shown in Figure 9.

From above results, the fulvic acid is effective for the removal of heavy metal ions. With the treatment of electro-osmosis, the effect of organics on the migration of heavy metal ions is also proved by some researchers [55,56,57,58]. Qian [56] used fulvic acid as a chelating agent to remove Pd^2+^ ions from the soil during electro-osmosis. The results showed that the incorporation of 0.5 mol/L of fulvic acid could result to a lead removal rate of 40.9% in the anode region. An et al. [57] added an acetic acid solution with a pH of 3.5 in the electro-kinetic repair of Cu^2+^ and Zn^2+^ contaminated kaolin, and the results showed that the removal rate of Cu^2+^ and Zn^2+^ ions reached 30%~80%. Zhao et al. [58] set an activated carbon PRB in the reaction zone, and used citric acid-sodium citrate as a buffer to electrically repair the composite heavy metal contaminated kaolin. Finally, the average removal rates of Cu^2+^, Cd^2+^, and Pb^2+^ ions were 39.93%, 99.42%, and 39.36%, respectively.

**Figure 10:**
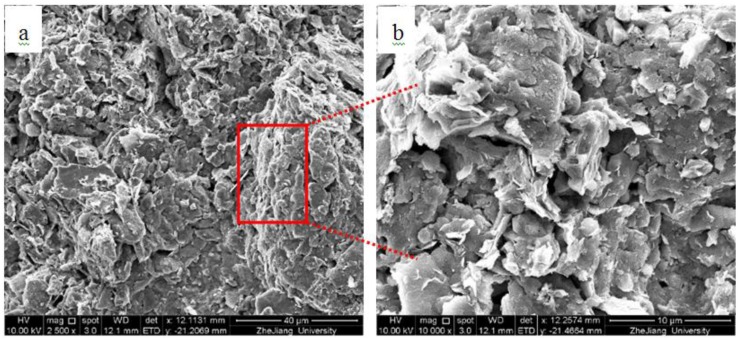

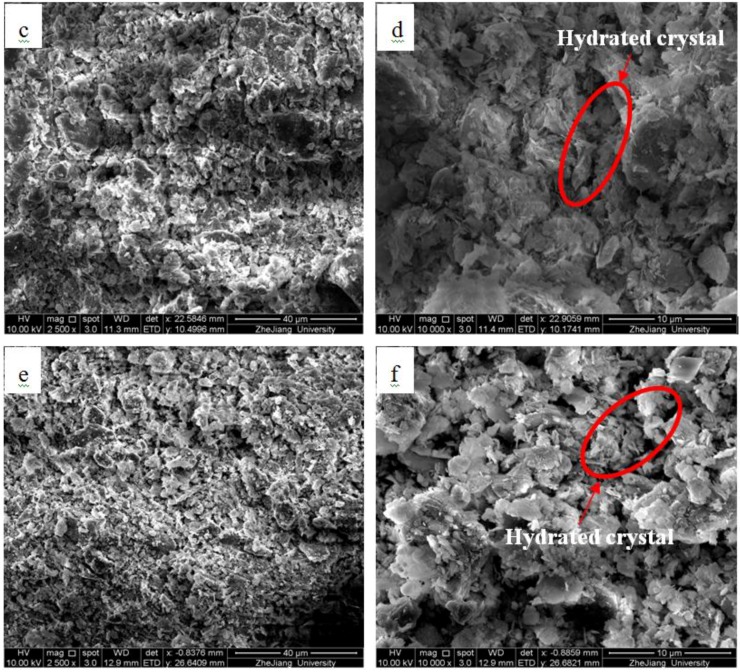
SEM micrographs of solidified sediment with different magnification, (**a**,**b**) natural sediment; (**c**,**d**) natural sediment mixed with 8% curing agent; (**e**,**f**) natural sediment mixed 3% organics and 8% curing agent.

Due to the different experiment methods, the maximum removal rate of Cu^2+^ and Zn^2+^ ions at the anode is 21% and 17%, respectively. Therefore, the method of electro-osmosis dewatering is suitable for repairing sediment with amount of heavy metals. As shown in Figure 9, the structure of fulvic acid is characterized as a loose assembly of aromatic organic polymers with many carboxyl groups (COOH) that release hydrogen ions, resulting in species that have electric charges at various sites on the ion. It is especially reactive with heavy metals, forming strong complexes with Zn^2+^, Al^3+^, and Cu^2+^ in particular, and leading to their increased solubility in natural waters. Fulvic acid is believed to originate as a product of microbial metabolism, although it is not synthesized as a life-sustaining carbon. Meanwhile, the fulvic acid in the sediment can enhance the migration ability of Cu^2+^ and Zn^2+^ ions through complexation, and effectively reduce the formation of colloids. Fulvic acid can effectively reduce the corrosion of the electrode during electro-osmosis, but the dewatering capacity of the sediment with a higher fulvic acid content is weaker. On the other hand, the fluvic acid would combine with Al^3+^ and Ca^2+^ (as shown in Figure 10).

In the following discussion, considering the defects of electro-osmosis in energy consumption and heavy metal ion migration [59,60], the solidification/stabilization method was used for improving the physical and engineering properties of problematic sediment to some predetermined targets [61,62,63], which has been widely utilized for the remediation of polluted soils.

### 3.5. Effect of Organics on the Strength of Solidified Sediment

The solidified soil was prepared by using the natural sediment with low organics content mixed with 8% stabilizers, including cementitious material and oxidant. Different levels of organics were compared with UCS.

Table 3 shows that as the fulvic acid content increases, the strength of the solidified sediment decreases. As the amount of fulvic acid increases from zero to 4.5%, the decrease in 7d strength of the solidified sediment becomes more obvious. However, with the increase of curing age, the strength growth rate of the A3 group is faster, and the 28d intensity difference of the solidified sediment is reduced. This shows the inhibition of solidification by organics—and the effect on the early strength of the solidified sediment is particularly significant.

As shown in Figure 10, natural sediment (a, b) is characterized by its flocculation structure, weak connection, and low strength. During the process of solidification (as shown in sample c, d, e, f), the microcrystalline substance in the sample increases, whereas on the contrary, the pore of solidified sediment decreases. Calcium silicate hydrate and calcium aluminate hydrate are produced in the solidified sediment, and the smaller soil particles form larger soil aggregate around the soil particle media. Moreover, the previous research indicated that the gel particles generated by hydration of cementitious material have strong adsorption activity [17], which can further bind larger soil aggregates to form the compact structure, and greatly improve the strength of the solidified sediment.

In addition, the formation of ettringite fills the pores in the soil, and the solid phase volume increases during the formation process, so as to compress the soil and improve the strength of the soil (c, d). When the concentration of CaO and OH^−^ in the pore water of solidified sediment is low, AFt is generated as a single crystal in the pore space. Due to its volume expansion, it fills the pore space. At the same time, its columnar crystals intersect with each other and form a unique spatial network structure together with C–S–H, which changes the pore distribution in the solidified sediment and makes the pore finer. The formation of AFt reduced the porosity of solidified sediment and the average pore diameter; therefore, it increases the strength of the solidified sediment [64].

From the micrographs of pores and hydration products, it can be inferred that organics have an influence on the inhibition of hydration reaction. The main reason is that fulvic acid can be combined with aluminum and calcium produced by cement hydration, delaying the progress of cement hydration, and simultaneously decomposing hydration products, hindering the hardening of cement, and thus affecting and destroying the formation of solidified soil structure and strength [18,19,20,21].

### 3.6. Effect of Organics and Curing Agents on Solidified Sediment

The solidified soil was prepared by using sediment and a different amount of curing agent and 3% organics. The relationship between the 7 d strength of the solidified sediment, the 28 d strength, and the amount of the curing agent is shown in Table 4. The strength of the solidified sediment test block is enhanced with the increase of the curing agent, and it is also confirmed that a certain amount of humic acid can inhibit the solidification of the sediment. Meanwhile, the effects of curing agents and organics have been compared and studied by many researchers [65,66,67]. It was found that the effect of cement combined with gypsum solidification was a significant improvement on that of single component [66,67]. Besides, in a study on the solidification of marine organics in the coastal areas of China, it was concluded that when the organics content is more than 3%, the organics have a great influence on the pile. However, when organics content exceeds 10%, it should be treated carefully when the cement was used to strengthen the foundation [66]. Lin [67] solidified sediment in cement with anhydrous gypsum, indicating that anhydrite gypsum mixed with 10–15% of cement by weight can improve the early strength of solidified sediment. According to the mechanism of fulvic acid in the process of solidification of sediment, the increasing strength of the solidified sediment is mainly due to decomposition of fulvic acid.

## 4. Conclusions

This study combined electro-osmotic dewatering and electric repair to treat river sediment, using the same equivalent voltage to carry out dewatering reinforcement and heavy metal Cu^2+^, Zn^2+^ ion reduction repair. Meanwhile, the effects of the organics content on the characteristics of the solidified sediment were investigated. From the above research results, several conclusions can be summarized as follows:

- Fulvic acid can effectively reduce the corrosion of the electrode during electro-osmosis, but the addition of fulvic acid under a constant current results in a decrease in the dewatering rate of electro-osmosis. The energy consumption coefficient of the electroosmotic process in this study is controlled by the equivalent voltage, regardless of the type of electrolyte added. In addition, the experiment revealed that the gravity field still plays an important role in simulating one-dimensional horizontal dewatering electro-osmosis.
- The heavy metals (Zn^2+^, Cu^2+^) have less effect on the displacement and dewatering rate of sediment with electro-osmosis treatment, and the fulvic acid in the sediment can enhance the migration ability of Cu^2+^ and Zn^2+^ ions through complexation. In addition, the experimental study shows that the average reductions of Cu^2+^ ions and Zn^2+^ ions are 28 and 11 mg/kg, respectively, which indicates the migration capacity of Cu^2+^ ions in sediment is stronger than that of Zn^2+^ ions.
- The experimental results show that fulvic acid in sediment can reduce the adsorption between Cu^2+^, Zn^2+^ ions and soil particles through complexation, enhance the migration ability of Cu^2+^, Zn^2+^ ions, and effectively reduce the formation of colloids. Fulvic acid can effectively reduce the corrosion of electrodes during electro-osmosis; meanwhile, the sediment containing a high content of fulvic acid has a stronger plasticity and a weaker electro-osmosis dewatering capacity compared with ordinary sediment. Therefore, electro-osmosis treatment is not recommended for sediment with a high content of fulvic acid.
- The electro-osmosis repair method has a better reinforcement effect on river sediment, but the migration of Cu^2+^ and Zn^2+^ ions in the short-time energization process is limited, so it is necessary to combine solidification treatment for heavy metal contained sediment.
- During the solidification process of river sediment, the fulvic acid in organics can be combined with aluminum and calcium produced by cement hydration, delaying the progress of cement hydration, simultaneously decomposing hydration products, hindering the hardening of cement, and thus affecting and destroying the formation of solidified sediment structure and strength.

## Figures and Tables

**Table 1:** Properties of remolded soil.

Relative Density *G*_s_	Density *ρ* (g·cm^−3^)	Initial Water Content *w*_0_ (%)	Plastic Limit *w*_p_ (%)	Liquid Limit *w*_L_ (%)	pH	Cu^2+^ (mg/kg)	Zn^2+^ (mg/kg)
2.64	1.36	68	30	42	7.26	10	20

**Table 2:** Experiment scheme for heavy metal ion electroosmotic migration.

Sample	Electricity (A)	Cu^2+^ (mg/kg)	Zn^2+^ (mg/kg)	Fulvic Acid Dosage (%)
C38F0	0.2	380	0	0
Z38F0	0.2	0	380	0
C19Z19	0.2	190	190	0
C38F45	0.2	380	0	4.5
Z38F45	0.2	0	380	4.5
E2F45	0.2	190	190	4.5
E0F45	0	190	190	4.5
C0Z0	0.2	0	0	4.5

**Table 3:** Strength of sludge solidified sediment with different organics content.

Sample	Fulvic Acid/%	7 d Intensity/MPa	28 d Intensity/MPa
F0	0	0.35	0.58
F15	1.5	0.28	0.45
F3	3	0.13	0.39
F45	4.5	0.04	0.25

**Table 4:** Strength of river sediment solidified with different dosages.

Sample	Curing Agent Dosage/%	Organics Content/%	7d/MPa	28d/MPa
C3	3	0	0.11	0.18
C5	5	0	0.18	0.34
C5O3	5	3	0.10	0.26
C8	8	0	0.35	0.58
